# Understanding Sports-Related Health Issues

**DOI:** 10.3390/jfmk9030136

**Published:** 2024-08-09

**Authors:** Daniel Rojas-Valverde

**Affiliations:** 1 Centro de Investigación y Diagnóstico en Salud y Deporte (CIDISAD), Escuela de Ciencias del Movimiento Humano y Calidad de Vida, Universidad Nacional, Heredia 86-3000, Costa Rica; drojasv@una.cr; Tel.: +506-88250219; 2 Clínica de Lesiones Deportivas (Rehab&Readapt), Escuela de Ciencias del Movimiento Humano y Calidad de Vida, Universidad Nacional, Heredia 86-3000, Costa Rica

In general, health problems (e.g., mental health issues, injuries, and renal, cardiovascular, respiratory, and integumentary problems) stemming from sports are undeniable at a wide variety of ages and athletes’ fitness levels. Yet, through an interdisciplinary approach, researchers and practitioners can address these issues and apply effective strategies to tackle them. This Special Issue, “Understanding Sports-Related Health Issues”, aims to contribute to this understanding, promote athletes’ health and well-being, and provide a comprehensive overview of the latest research on sports-related health issues.

Understanding these issues is vital for ensuring the health of athletes and individuals engaging in physical activities. These potential health problems encompass a wide range of conditions, from acute injuries, such as sprains and fractures, to chronic issues, such as overuse injuries and degenerative joint diseases [1]. Additionally, mental health concerns, including performance anxiety and depression, are increasingly recognized as significant factors affecting athletes’ overall health and performance [2].

To comprehensively address these issues, an interdisciplinary approach is essential. This involves collaboration between researchers, healthcare professionals, coaches, and athletes. Researchers contribute by conducting studies to identify risk factors, mechanisms of injury, and effective prevention strategies. Healthcare professionals, including physicians, physical therapists, nutritionists, and sports psychologists, play a crucial role in diagnosing and treating sports-related injuries and mental health issues [3]. Coaches are essential in implementing injury prevention programs, monitoring athletes’ workload, and promoting a culture of safety and well-being within sports teams.

Effective strategies for preventing and managing sports-related health issues involve a combination of education, training, and policy initiatives. Athletes should receive proper education on injury prevention techniques, including proper warm-up, technique correction, and the gradual progression of training intensity. Coaches and sports organizations should implement evidence-based protocols for injury prevention, such as neuromuscular training programs and rule modifications, to reduce contact-related injuries.

This Special Issue compiles fourteen studies in different areas. Regarding exercise intensity preferences and behavioral intervention, one study (Contribution 1) explores the relationship between exercise intensity preferences, tolerance, competence satisfaction, and frustration within fitness settings, revealing their impact on exercise intentions. Positive correlations were found, with exercise intensity preference positively linked to competence satisfaction and negatively associated with competence frustration, indirectly influencing exercise intentions. Additionally, a separate investigation (Contribution 2) examines the effects of sprint interval training (SIT) on explosive muscle force production in female high school field lacrosse players, demonstrating significant improvements in the rate of torque development, contractile impulse, and muscle function following a 12-week SIT regimen concurrent with field lacrosse-specific training. These findings suggest that integrating SIT into training regimens may enhance athletes’ performance on the field, emphasizing the importance of tailored exercise interventions in optimizing sports-related health outcomes.

Two studies are included on exercise and mental health-related issues. A pilot study (Contribution 3) examines the impact of peer-supported versus self-guided exercise interventions on anxiety and depression levels among young adults. In a randomized parallel group design involving 27 participants over eight weeks, both interventions reduced anxiety and depression scores, though decay was more pronounced in the peer-supported group. Self-guided exercise showed longer-lasting effects, suggesting it could be a cost-effective approach to addressing mental health issues in this demographic. Meanwhile, managing exercise-induced asthma in athletes involves understanding its complex pathogenesis, varied diagnostic methods, and tailored treatments. Optimal management enhances athletes’ quality of life and performance, emphasizing personalized care for respiratory conditions in sports (Contribution 4).

There is a strong relationship between nutrition and supplementation for exercise-related injuries. The first study (Contribution 5) used a cherry tart supplement to explore its impact on exercise-induced muscle damage recovery. Despite supplementing 1000 mg for eight days, no significant effects were found on muscle function, soreness, or activation in seventeen women completing an intense leg exercise protocol. Meanwhile, a study on male Army cadets (Contribution 6) revealed concerning trends: 62% exhibited low energy availability, none met optimum thresholds, and dietary intake fell short of military dietary reference intakes. Additionally, 85% experienced poor sleep quality. While low energy availability was correlated with higher fat mass, no direct link was found with sleep quality, underscoring the need for tailored interventions to optimize health and performance in military settings.

In addition, pain is associated with numerous health problems. One study (Contribution 7) included in this Special Issue explores the link between pain vigilance, memory of pain, and current injury-related pain with kinesiophobia in athletes from Iran and the United States who experienced serious injuries affecting their sports participation. Findings from 172 athletes show that heightened pain vigilance and memory of past pain are positively associated with kinesiophobia, explaining 31% of the variance in scores. These results suggest that excessive focus on pain-related cues and lingering memories of past injuries contribute to fear of movement and reinjury in current and former athletes. Additionally, stretching and releasing techniques are commonly used in managing iliotibial band syndrome, though evidence for their efficacy remains inconclusive, necessitating further research for optimal treatment strategies (Contribution 8).

Another topic in this issue includes the analysis of gender differences and metabolism (Contribution 9). The study investigated sex differences in resting metabolic rate (RMR) among collegiate athletes and its correlation with body composition parameters. While men exhibited higher absolute RMR than women, adjustments for body and fat-free mass made the differences insignificant. Body mass emerged as the strongest predictor of RMR in both genders. Concurrently, a comparison between basketball and soccer players’ knee strength profiles during puberty found similar development patterns in isokinetic strength, with basketball players consistently demonstrating higher absolute peak torque values but no differences when normalized to body mass (Contribution 10). These findings shed light on physiological nuances in athletes’ metabolic and muscular adaptations across sports and genders.

Performance assessment and data analysis are critical for preventing and treating health problems in sports. Four studies regarding this topic are included in this Special Issue. Biological age significantly influences the development of speed, agility, and explosive power in young tennis players, as revealed in a study involving fifty participants (Contribution 11). Players with post-peak height velocity (PHV) displayed superior performance across various tests compared to their pre-PHV counterparts, showcasing advantages in explosive power and sprinting abilities. These findings underscore the importance of considering biological age when evaluating athletic potential and designing training regimens for young tennis players. Monitoring youth squads during a national tournament highlighted distinct performance metrics between under-15 and under-19 players in rugby sevens (Contribution 12). While both groups exhibited high-intensity running and sprinting, younger players experienced greater overall workload and sprint rates. Understanding these differences can help coaches tailor training programs to suit the specific physiological needs of youth rugby players, optimizing player development and minimizing the risk of injury. Additionally, qualitative insights into coaching practices in bodybuilding revealed consistency in recommendations regarding nutrition, exercise protocols, supplements, and even performance-enhancing drugs, underscoring the prevalence of certain strategies within the bodybuilding community (Contribution 13). Lastly, a systematic review of case studies in physique athletes during contest preparation revealed significant alterations across various physiological and psychometric measures, highlighting the complex interplay between training, nutrition, and hormonal adaptations in pursuing competitive success (Contribution 14).

## Conclusions

In conclusion, addressing sports-related health issues requires an interdisciplinary approach where researchers, healthcare professionals, coaches, and athletes collaborate to understand and mitigate potential risks. The Special Issue, “Understanding Sports-Related Health Issues”, contributes to this effort by compiling fourteen studies covering various topics. From exploring exercise intensity preferences and behavioral interventions to analyzing gender differences in metabolism, each study sheds light on critical aspects of athlete health and performance. These insights inform tailored interventions, such as injury prevention protocols and mental health support, to optimize athletes’ well-being and performance. The studies underscore the importance of personalized care and evidence-based strategies for effectively managing sports-related health issues. By embracing a comprehensive approach, stakeholders can enhance athletes’ health, safety, and success across various disciplines.
